# Clonal expansion and linear genome evolution through breast cancer progression from pre-invasive stages to asynchronous metastasis

**DOI:** 10.18632/oncotarget.3111

**Published:** 2015-01-29

**Authors:** Anne Bruun Krøigård, Martin Jakob Larsen, Anne-Vibeke Lænkholm, Ann S. Knoop, Jeanette D. Jensen, Martin Bak, Jan Mollenhauer, Torben A. Kruse, Mads Thomassen

**Affiliations:** ^1^ Department of Clinical Genetics, Odense University Hospital, 5000 Odense C, Denmark; ^2^ Human Genetics, Institute of Clinical Research, University of Southern Denmark, 5000 Odense C, Denmark; ^3^ Department of Pathology, Slagelse Hospital, 4200 Slagelse, Denmark; ^4^ Department of Oncology, Rigshospitalet, 2100 Copenhagen, Denmark; ^5^ Department of Oncology, Odense University Hospital, 5000 Odense C, Denmark; ^6^ Department of Pathology, Odense University Hospital, 5000 Odense C, Denmark; ^7^ Lundbeckfonden Center of Excellence NanoCAN, 5000 Odense C, Denmark; ^8^ Molecular Oncology Group, Institute of Molecular Medicine, University of Southern Denmark, 5000 Odense C, Denmark

**Keywords:** Breast cancer, Metastasis models, Copy number aberrations, Linear progression model

## Abstract

Evolution of the breast cancer genome from pre-invasive stages to asynchronous metastasis is complex and mostly unexplored, but highly demanded as it may provide novel markers for and mechanistic insights in cancer progression. The increasing use of personalized therapy of breast cancer necessitates knowledge of the degree of genomic concordance between different steps of malignant progression as primary tumors often are used as surrogates of systemic disease. Based on exome sequencing we performed copy number profiling and point mutation detection on successive steps of breast cancer progression from one breast cancer patient, including two different regions of Ductal Carcinoma *In Situ* (DCIS), primary tumor and an asynchronous metastasis. We identify a remarkable landscape of somatic mutations, retained throughout breast cancer progression and with new mutational events emerging at each step. Our data, contrary to the proposed model of early dissemination of metastatic cells and parallel progression of primary tumors and metastases, provide evidence of linear progression of breast cancer with relatively late dissemination from the primary tumor. The genomic discordance between the different stages of tumor evolution in this patient emphasizes the importance of molecular profiling of metastatic tissue directing molecularly targeted therapy at recurrence.

## INTRODUCTION

Breast cancer progression results from stochastic events leading to the acquisition of genomic alterations resulting in reduced apoptosis, replicative immortality, evasion of growth suppressors, uncontrolled proliferation, reprogrammed energy metabolism, evasion of immune destruction, angiogenesis, invasion and metastasis [1]. Approximately 500 genes are known to be involved in carcinogenesis [2], but relatively little is known about the genes driving metastatic progression. Metastases represent the final products of a multi-step biological process as the metastatic cascade involves many critical steps, which are still poorly understood. Different genes are believed to be involved at different stages, as the metastatic process poses very diverse challenges to the cell, including detachment, motility, invasion, survival in circulation, extravasation, adaptation to a new environment and organ-specific colonization [3]. Cancer progression may be regarded as a process of natural selection, where genomic alterations conferring a selective advantage for the cell in a given environment and time point of the progression process as well as selection pressures provided by treatment results in the formation of the most aggressive clones.

Monoclonal origin of cancer, as proposed by Nowell in 1976 [4] is widely accepted. However, controversy exists between two fundamental models of malignant progression, addressing the issue of the timing of metastasis-enabling genomic alterations and the degree of genomic concordance between primary tumors and its metastases. According to the linear progression model, the malignant cells pass through multiple successive rounds of genetic changes and selection within the primary tumor microenvironment, before tumor cell dissemination successfully results in a metastatic lesion. From this perspective, metastases are seeded by the most advanced and aggressive clone that should also dominate the primary tumor [5]. The parallel progression model proposes parallel, independent progression of metastases arising from early disseminated tumor cells and predicts greater disparity between the primary tumor and metastatic lesions. The model emphasizes independent accumulation of genetic and epigenetic alterations as the metastasis is subject to site-specific selection pressures [6].

Somatic copy number alterations and point mutations contribute to malignant progression, by altering the expression or functions of cancer driver genes. DNA breakpoints are non-randomly distributed and breakpoint hotspots are influenced by chromatin architecture [7], replication timing [8], specific repeat sequences, G-quadruplex sequences and hypomethylation [9]. Distinct patterns have been found for common cancer breakpoint hotspots and cancer-type-specific breakpoint hotspots [10]. Somatic copy number events can accumulate progressively or result from punctuated bursts of evolution in catastrophic events like chromothripsis [11]. Oncogene amplification can take place either in double minute chromosomes or intrachromosomally through breakage-fusion-bridge cycles [12].

We set out to illuminate some of the unresolved issues of breast cancer progression. It is unknown when actually the metastasis founder cell leaves the primary tumor and how similar early and late stages of breast cancer progression are at the genetic level. Another unresolved aspect is the degree of clonal diversity within cancer tissue. Furthermore, it has been discussed whether genetic aberrations accumulate gradually over time or result from catastrophic events. A very limited number of studies have included breast cancer samples separated by both space and time, which is needed in order to address such questions.

Using exome sequencing and validation using targeted deep sequencing we conducted genome-wide copy number profiling and point mutation detection on successive steps of breast cancer progression from one patient, who had received neo-adjuvant and subsequently adjuvant treatment, including two regions of pre-invasive tissue, primary tumor and an asynchronous metastasis. We report limited clonal heterogeneity, possibly involving catastrophic events and substantial genomic discordance between early tumor stages and the asynchronous metastasis with data in favor of a linear progression model.

## RESULTS

### Somatic events are retained throughout breast cancer progression

To investigate the genome evolution through breast cancer progression, successive tumor samples from an estrogen receptor positive, HER2-negative breast cancer patient undergoing mastectomy and an asynchronous metastasis were collected and thoroughly analyzed using next generation sequencing. The patient had initially been treated with five series of neo-adjuvant Cyclophosphamide, Epirubicin and Fluorouracil (CEF). From the mastectomy specimen two topologically separated regions of Ductal Carcinoma *in Situ* (DCIS) and a primary tumor region were secured. In addition to the neo-adjuvant chemotherapy, all tumor samples included in the study had been subject to endocrine treatment with Tamoxifen or Anastrozole. Following mastectomy, the patient received four series of Taxotere/Gemcitabine and radiation therapy. In spite of the extensive therapy and ongoing endocrine treatment, the patient experienced recurrence after 4.05 years and from a contralateral periclavicular lymph node an asynchronous metastasis was biopsied and included in the study. Exome sequencing was performed on DNA from each tumor sample and somatic copy number mutations were detected by two supplementing analyses. The pseudo-CGH ngCGH, using simple coverage counting on tumor sequencing reads relative to normal reads, plottet in Log2 ratios, reveal copy number imbalances between tumor tissue and matched normal tissue. These genomic quantity measurements of tumor sequencing data relative to normal, were supported by a plot of B Allele Frequencies (BAFs) measuring the allelic imbalance in the tumor tissue. These BAFs are not displaying the usual variant allele frequency, as they include only positions with known heterozygote SNPs of the patient. Hence, these BAFs support copy number events by providing information about the fraction of sequenced cells in the cancer sample to be affected by a somatic copy number event, and enable detection of subclonality within the cancer cell population. For a detailed description of the ngCGH technique and the accompanying BAFs, see the methods section.

**Figure 1:**
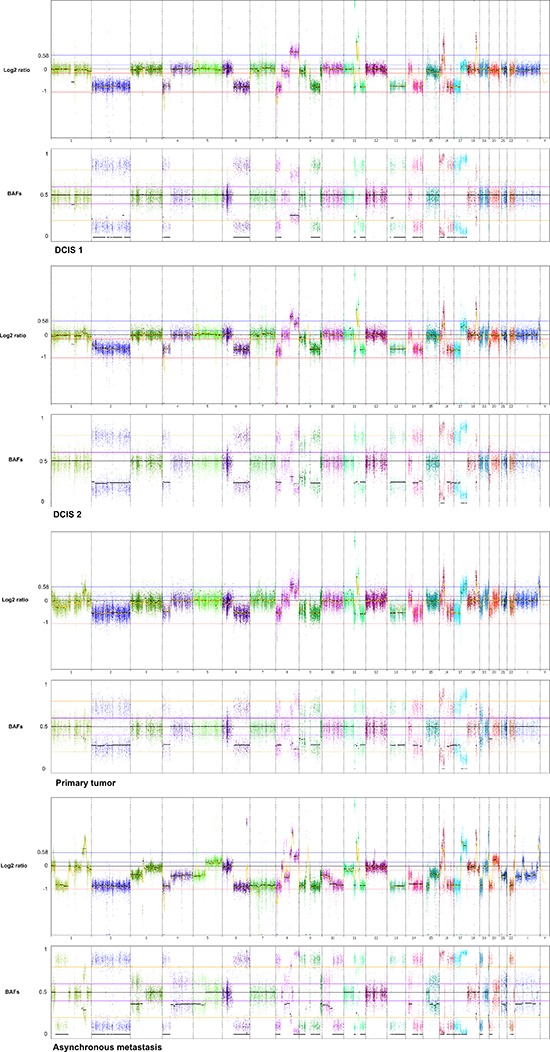
Genome wide displays of copy number mutation data Log2 ratios and corresponding B Allele Frequencies (BAFs) are in upper and lower panels, respectively of each tumor sample. The Log2 ratios constitute a genomic quantity measurement of the tumor sample relative to the normal. The BAF plots are based on known heterozygote SNP positions of the patient, thus depicting the allelic imbalances of the tumor sample.

Genome-wide displays of Log2 ratios and corresponding BAFs from each step of malignant progression are displayed in Figure 1. Our results reveal overall striking similarities in copy number patterns between different steps of cancer evolution in the studied patient. In Table 1 all copy number events and the concordance of the aberrations between the samples are listed. All steps of malignant progression display Loss of Heterozygosity (LOH) of the entire chromosome 2, which is also seen in the BAF plot, confirming that all of the malignant cells have lost one of the alleles. Similarly, copy number losses on 4p, 6q, 8p, 9q, 11q, 13p-q, 14q, 16p, 16q, 17p, 17q, 19q and 21q are supported by the BAFs and are present in all samples. Overview of copy number aberrations in all the samples are provided in Supplementary Figures 1–4. Chromosome 8, 11 and 16 are subjects to widespread amplifications, but whereas the events on chromosome 11 and 16 are a common trait of this cancer genome, chromosome 8q undergoes additional amplification during breast cancer progression. All samples display high copy number gain at 8q21.3–24.3, containing the known oncogenic driver *MYC* (8q24.21), but additional events arise in the later stages, and thus chromosome 8 appears repeatedly in Table 1. In total, 36 copy number events were identified in DCIS 1, comprising 25 loss events and 11 gain events, as seen in Table 1. The exact localization of breakpoints found in DCIS 1 are retained in all later stages of tumor progression, confirming common ancestry of the malignant cells of progression.

**Table 1:** Copy number events within four steps of malignant progression The x informs whether the event is present within each tumor sample. LOH: loss of heterozygosity. HL: homozygote loss. OCL: one copy loss. CG: Copy gain. HCG: high copy gain. VHCG: very high copy gain. EHCG: extremely high copy gain. *denotes that for copy gain events it is not possible to discriminate between copy gain within all the malignant cells or higher amplification within a subclone of cells

Chr	Start (Mb)	End (Mb)	Length (Mb)	Cytobands	Type	# genes	DCIS 1	DCIS 2 complete	DCIS 2 subclone	Primary tumor complete	Primary tumor subclone	Metastasis complete	Metastasis subclone
**2**	0	243.19	243.19	p25.3–q37.3	LOH	1545	x	x	x	x	x	x	x
**4**	0	49.53	49.53	p16.3–p11	LOH	346	x	x	x	x	x	x	x
**6**	68.64	160.13	91.48	q12–q25.3	LOH	439	x	x	x	x	x	x	x
**8**	0	37.68	37.68	p23.3–p11.23	LOH	306	x	x	x	x	x	x	x
**8**	7.04	8.06	1.02	p23.1	HL	46	x	x	x	x	x	x	x
**8**	11.88	12.42	0.54	p23.1	HL	18	x	x	x	x	x	x	x
**8**	39.44	41.05	1.61	p11.22–11.21	LOH	6	x	x	x	x	x	x	x
**9**	68.18	130.82	62.64	q13–q34.11	LOH	470	x	x	x	x	x	x	x
**11**	62.41	68.28	5.86	q12.3–q13.2	LOH	240	x	x	x	x	x	x	x
**11**	71.11	75.90	4.79	q13.4–q13.5	LOH	79	x	x	x	x	x	x	x
**11**	85.72	87.59	1.87	q14.2	LOH	11	x	x	x	x	x	x	x
**11**	100.81	135.00	34.19	q22.1–q25	LOH	322	x	x	x	x	x	x	x
**13**	16.48	115.16	98.68	p11.1–q34	LOH	518	x	x	x	x	x	x	x
**14**	43.95	80.13	36.17	q21.2–q31.1	LOH	287	x	x	x	x	x	x	x
**14**	84.27	106.74	22.47	q31.2–q32.33	LOH	285	x	x	x	x	x	x	x
**16**	0.08	1.4	1.31	p13.3	LOH	65	x	x	x	x	x	x	x
**16**	3.04	4.48	1.44	p13.3	LOH	46	x	x	x	x	x	x	x
**16**	24.56	27.43	2.87	p12.1	LOH	16	x	x	x	x	x	x	x
**16**	32.60	33.37	0.77	p11.2	LOH	11	x	x	x	x	x	x	x
**16**	33.55	34.46	0.90	p11.2	LOH	3	x	x	x	x	x	x	x
**16**	45.61	90.35	44.74	q11.2–q24.3	LOH	446	x	x	x	x	x	x	x
**17**	0	22.59	22.59	p13.3–p11.1	LOH	414	x	x	x	x	x	x	x
**17**	25.29	41.45	16.16	q11.1–q21.31	LOH	400	x	x	x	x	x	x	x
**19**	57.79	59.12	1.33	q13.43	LOH	66	x	x	x	x	x	x	x
**21**	45.68	48.12	2.44	q22.3	LOH	63	x	x	x	x	x	x	x
**8**	112.88	146.36	33.47	q23.3–q24.3	OCL	235			x	x	x	x	x
**9**	0	44.41	44.41	p24.3–p11.2	LOH	283			x	x	x	x	x
**20**	0	11.90	11.90	p13–p12.2	LOH	136			x	x	x	x	x
**1**	26.08	107.60	81.51	p36.11–p13.3	LOH	717				x	x	x	x
**20**	31.89	32.83	0.93	q11.21–11.22	LOH	15				x	x	x	x
**22**	16.39	42.11	25.72	q11.1–q13.2	LOH	433				x	x	x	x
**22**	42.45	42.77	0.31	q13.2	LOH	9				x	x	x	x
**19**	0.03	20.88	20.85	p13.3–p12	LOH	648					x	x	x
**20**	11.93	21.5	9.56	p12.2–p11.22	LOH	55					x	x	x
**10**	65.05	135.53	70.47	q21.3–q26.3	LOH	608						x	x
**3**	0	76.53	76.53	p26.3–p12.3	LOH	592							x
**4**	50.40	191.15	140.75	q11–q35.2	LOH	664							x
**5**	0	77.46	77.46	p15.33–q14.1	LOH	395							x
**7**	0	159.13	159.13	p22.3–q36.3	HL	1190							x
**8**	52.54	53.33	0.78	q11.22–11.23	LOH	3							x
**8**	55.37	67.35	11.98	q11.23–q13.1	LOH	59							x
**8**	68.34	86.56	18.22	q13.2–q21.2	LOH	76							x
**9**	130.73	141.21	10.48	q34.11–q34.3	LOH	252							x
**10**	0	65.05	65.05	p15.3–q21.3	LOH	387							x
**14**	18.80	42.09	23.28	q11.2–q21.1	LOH	227							x
**15**	19.96	42.13	22.16	q11.1–q15.1	HL	323							x
**15**	42.16	102.53	60.36	q15.1–q26.3	LOH	590							x
**21**	9.74	45.93	36.19	p11.2–q22.3	LOH	270							x
**X**	0	42.79	42.79	p22.33–p11.3	LOH	209							x
**X**	55.11	60.60	5.48	p11.21–q11.1	LOH	20							x
**X**	61.69	102.34	40.65	q11.1–q22.1	LOH	198							x
**X**	106.50	132.09	25.58	q22.3–q26.2	LOH	230							x
**Copy number gain events**													
**8**	87.65	146.36	58.71	q21.3–q24.3	HCG	359	x	x	x	x	x	x	x
**11**	68.28	71.11	2.82	q13.2–q13.4	EHCG	26	x	x	x	x	x	x	x
**11**	75.90	85.33	9.43	q13.5–q14.1	VHCG	44	x	x	x	x	x	x	x
**11**	88.30	89.46	1.16	q14.3	CG	5	x	x	x	x	x	x	x
**11**	89.53	94.56	5.02	q14.3–q21	VHCG	46	x	x	x	x	x	x	x
**16**	1.40	2.79	1.39	p13.3	CG	80	x	x	x	x	x	x	x
**16**	15.50	18.43	2.93	p13.11–p12.3	CG	29	x	x	x	x	x	x	x
**16**	18.81	20.38	1.57	p12.3	CG	18	x	x	x	x	x	x	x
**16**	20.80	24.56	3.76	p12.3–p12.1	VHCG	50	x	x	x	x	x	x	x
**16**	27.67	31.99	4.32	p12.1–p11.2	VHCG	154	x	x	x	x	x	x	x
**18**	55.39	63.42	8.03	q21.31–q22.1	VHCG	42	x	x	x	x	x	x	x
**17**	41.44	43.34	1.89	q21.31	CG	58		x^*^	x^*^	x	x	x	x
**17**	44.23	81.19	36.96	q21.31–q25.3	CG	542		x^*^	x^*^	x	x	x	x
**19**	53.65	56.85	3.20	q13.42–q13.43	CG	181		x^*^	x^*^	x	x	x	x
**X**	142.23	144.43	2.10	q27.3–q28	CG	5		x^*^	x^*^	x	x	x	x
**X**	146.55	151.56	5.00	q27.3–q28	CG	45		x^*^	x^*^	x	x	x	x
**1**	187.21	192.62	5.41	q31.1–q31.2	HCG	6				x^*^	x^*^	x	x
**1**	192.62	197.10	4.47	q31.2–q31.3	CG	18				x^*^	x^*^	x	x
**1**	197.10	215.74	18.64	q31.3–q41	HCG	202				x^*^	x^*^	x	x
**1**	215.74	218.50	2.75	q41	CG	7				x^*^	x^*^	x	x
**20**	21.37	26.06	4.98	p11.22–p11.1	CG	47				x^*^	x^*^	x	x
**20**	33.71	52.66	18.95	q11.22–q13.2	CG	222				x^*^	x^*^	x	x
**22**	46.66	50.65	3.98	q13.31–q13.33	HCG	29				x^*^	x^*^	x	x
**X**	144.43	146.55	2.12	q27.3	CG	30				x^*^	x^*^	x	x
**X**	151.56	155.05	3.49	q28	CG	133				x^*^	x^*^	x	x
**1**	218.50	219.37	0.86	q41	HCG	5						x^*^	x^*^
**6**	150.76	152.61	1.84	q25.1–q25.2	VHCG	9						x^*^	x^*^
**8**	49.26	51.67	2.40	q11.21	VHCG	6						x^*^	x^*^
**8**	51.67	52.54	0.86	q11.21–q11.22	CG	2						x^*^	x^*^
**8**	53.34	55.37	2.03	q11.23	VHCG	10						x^*^	x^*^
**8**	67.35	68.34	0.98	q13.1–q13.2	VHCG	17						x^*^	x^*^
**8**	100.58	111.46	10.87	q22.2–q23.2	EHCG	53						x^*^	x^*^
**12**	57.92	58.32	0.39	q13.3–q14.1	CG	22						x^*^	x^*^
**17**	46.04	46.57	0.52	q21.32	VHCG	8						x^*^	x^*^
**17**	47.36	81.08	33.71	q21.32–q25.3	VHCG	469						x^*^	x^*^
**19**	53.65	56.85	3.20	q13.42–q13.43	VHCG	181						x^*^	x^*^
**20**	29.89	31.89	1.99	q11.21	CG	47						x^*^	x^*^
**22**	46.66	50.65	3.98	q13.31–q13.33	VHCG	29						x^*^	x^*^
**X**	43.26	45.24	1.97	p11.3	CG	8						x^*^	x^*^
**X**	45.59	51.07	5.48	p11.3–p11.22	CG	157						x^*^	x^*^
**X**	51.64	54.31	2.67	p11.22	CG	67						x^*^	x^*^
**X**	142.32	144.43	2.11	q27.3	HCG	5						x^*^	x^*^
**X**	146.55	151.56	5.00	q27.3–q28	VHCG	45						x^*^	x^*^

**Figure 2:**
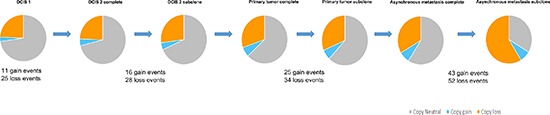
Copy number evolution of the studied cancer genome All aberrations from the previous steps in tumor progression are retained in the later stages.

The copy number evolution of the studied cancer genome is displayed in Figure 2. A total of 99.14 Mb, 148.29 Mb, 213.08 Mb and 239.55 Mb were copy gained and 786.27 Mb, 876.05 Mb, 1014.93 Mb and 1902.27 Mb were copy lost in DCIS 1, DCIS 2, primary tumor and asynchronous metastasis, respectively (Supplementary Table 2). As all aberrations are retained in later steps, 100% of the copy number events found in DCIS 1, DCIS 2 and primary tumor are found in the asynchronous metastasis. Of the 2141.82 Mb copy number events found in the asynchronous metastasis only 41.33% are present in DCIS 1. Of the 1228.01 Mb copy number events found in the primary tumor 72.10% are present in DCIS 1. Of the 1024.34 Mb copy number events found in DCIS 2 86.43% are present in DCIS 1. These concordance levels thus argue for a linear evolution of the analyzed cancer genome.

**Figure 3:**
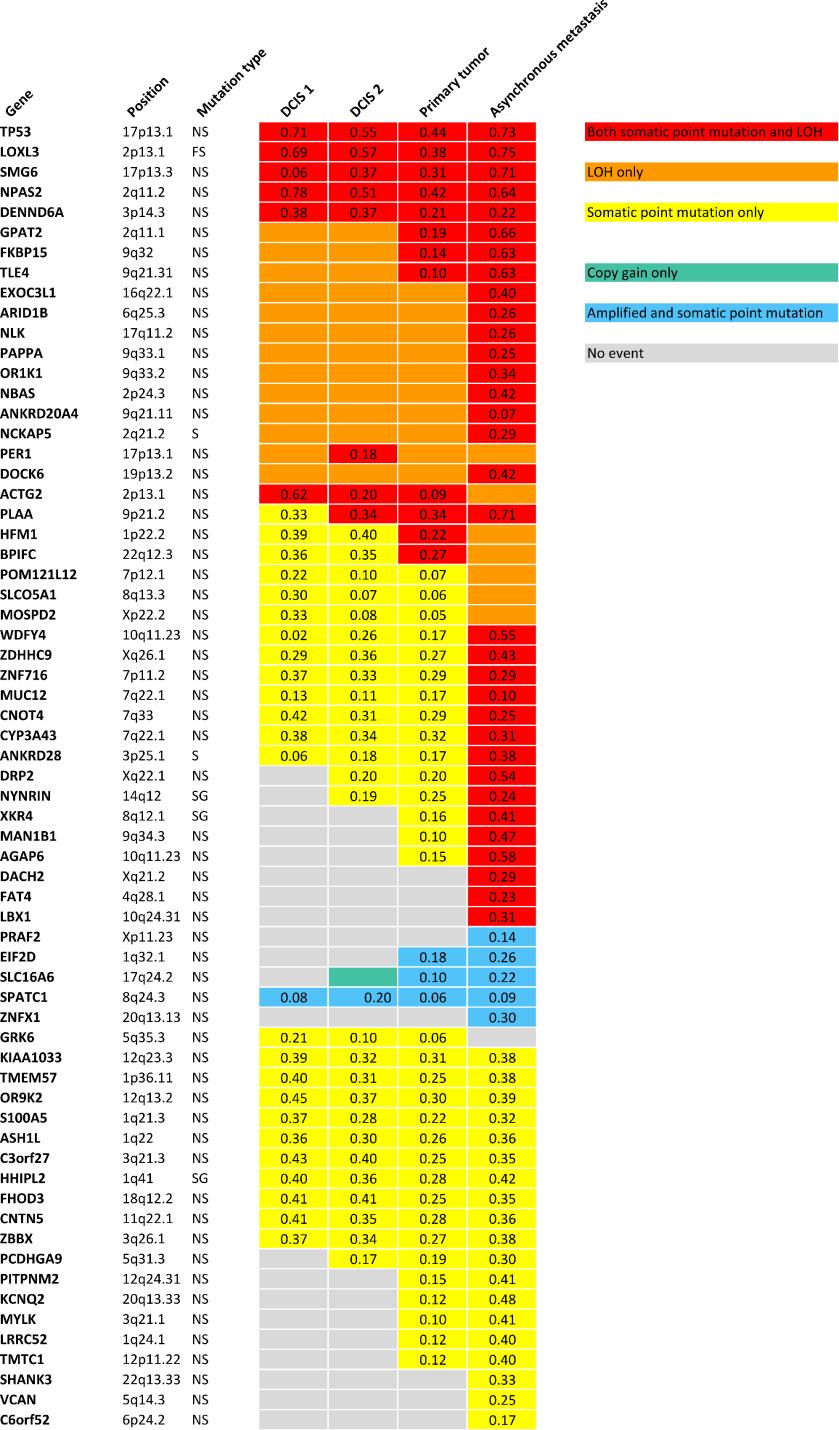
Validated point mutations specified with B Allele Frequencies combined with copy number events within each step of malignant progression NS: Nonsynonymous SNV. FS: Frameshift. S: Splicing. SG: Stopgain SNV.

**Figure 4:**
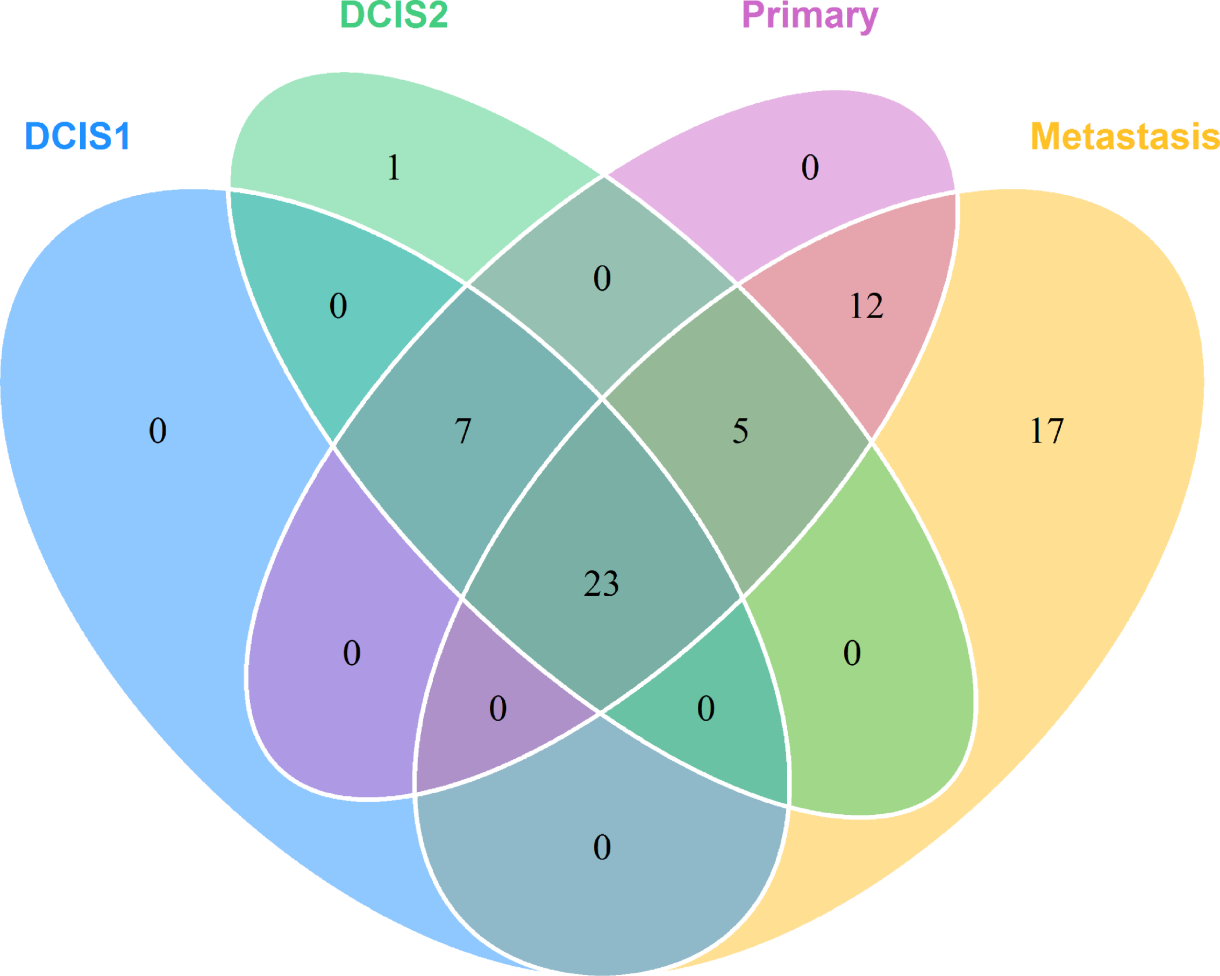
Venn diagram showing the mutational concordance of validated somatic mutations between the steps in malignant progression based on targeted deep sequencing

Somatic variant calling on the exome sequencing data provided 73 nonsynonymous, stopgain, splice and frameshift mutations, shown in Supplementary Table 3. To validate the mutations, targeted deep sequencing of the positions was performed. Sixtyfive point mutations could be validated. Validated point mutations combined with copy number events are displayed in Figure 3. Nucleotide changes, amino acid changes and functional prediction scores are displayed in Supplementary Table 4. A venn diagram of the mutational concordance between the samples is shown in Figure 4. Of the 65 validated point mutations detected, no mutations were private to DCIS 1 or the primary tumor while one mutation is exclusive to DCIS 2. Twenty-three mutations were shared by all steps of malignant progression and 17 were exclusively found in the metastasis.

### Subclonality within DCIS and evolution of somatic events

The DCIS 2 sample displays three copy number loss events and five copy number gain events not found in the other pre-invasive sample. All newly acquired aberrations remain throughout later stages of progression, again confirming the common ancestry between the later stages and this malignant clone. Copy number losses on chromosome 9p and 20p are subclonal events, supported by subclonal BAFs, illustrating that only a fraction of the malignant cells are affected by the loss event. For copy number gain events it is not possible to detect whether events are subclonal or complete events, as a region can be massively amplified within a subclone of cells or moderately gained in all malignant cells. The three copy number gain events on chromosome 19q and Xq may be subclonal events, which is suggested by BAFs not splitting out to the same extend as seen in the primary tumor and the metastasis.

### Subclonality within the primary tumor and subclonal origin of the metastatic cell

The primary tumor displays six additional copy number loss events and nine copy number gain events, solely shared between the primary tumor and the asynchronous metastasis, but not present in the pre-invasive tissue, suggesting that these events might contribute to invasiveness. Copy number losses on chromosome 19p and 20p are subclonal events in the primary tumor but a “pure” phenomenon in the asynchronous metastasis. Thus, a subclonal cell of the primary tumor was the one to succeed in forming the metastasis. The subclonal origin of the metastasis founder cell is further supported by point mutation data. The mutation frequencies in the *PITPNM2, KCNQ2, MYLK, LRRC52* and *TMTC1* genes are subclonal with BAFs in the primary tumor around 10–15% as seen in Figure 3 and are “purified” to comprise a complete heterozygote mutation frequency with BAFs around 40–48% in the metastasis.

### Excessive increase in somatic events and subclonality within the asynchronous metastasis

The asynchronous metastasis retains all previous aberrations and displays 18 copy number loss and 18 copy number gain events exclusive to the metastasis, amounting to a total of 52 loss events and 43 gain events, as shown in Table 1. Copy number events exclusively found in the metastasis might contain oncogenic driver genes of the metastatic process, e.g. the extreme amplification of chromosome 8q22.2–23.2, a small region containing 53 genes. Copy number discordant genes between the primary tumor and the metastasis are listet in Supplementary Tables 5 and 6.

**Figure 5:**
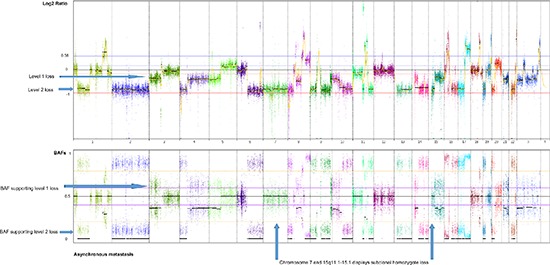
Genome-wide display of copy number events of the asynchronous metastasis The top panel displays Log2 ratios and the panel below displays BAFs of known SNPs heterozygote in the germline. Two prominent subclones within the asynchronous metastasis are revealed by two distinct levels of loss in the Log2 ratio plot, supported by the BAFs, displaying the fraction of cells participating in the event. Level 1 losses, originating from only a subclone of malignant cells, are accompanied by BAFs splitting out at an intermediate level. Level 2 losses, shared by all the malignant cells of the metastasis, are accompanied by BAFs splitting out to the level corresponding to a complete heterozygote loss.

Two major homogenous subclones coexist in the asynchronous metastasis, seen in the ngCHG data as two distinct levels of loss in the Log2 ratio panel and the fraction of cells involved in the loss event is supported by the BAFs, as seen in Figure 5. A Level 1 loss is subclonal, thus involves only a fraction of the malignant cells, while a Level 2 loss is complete as it originates from all of the malignant cells. One subclone retains all of the copy number aberrations from previous steps as well as an additional Level 2 loss on chromosome 10q, which likely constitutes an early event in the metastasis clone, as it is present in all of the metastatic cells. Another subclone contains all the aforementioned aberrations and 17 additional Level 1 losses. The existence of two distinct metastatic subclones are supported by the BAFs, as complete and subclonal losses are accompanied by BAFs splitting out completely towards 0 and 1 and intermediate BAFs, respectively. Chromosome 7 and chromosome 15q11.1–15.1 deviates from this rule as they display Level 2 losses accompanied by BAFs around 0.5. This phenomenon may be explained by a homozygote loss in one of the subclones of the metastasis, while the other subclone has retained the two alleles. The BAFs of 0.5 represents the allele distribution of the subclone retaining the two alleles. The homozygote loss of chromosome 7 and chromosome 15q11.1–15.1 reaches almost the same Log2 ratio loss level (Level 2) as the heterozygote loss levels of the entire distant metastasis, implying that the two subclones are approximately the same population size.

### Point mutations reveal potential drivers affected by both LOH and nonsynonymous mutation

Three of the exact point mutations, in the *TP53*, *NPAS2* and *MYLK* gene, are annotated to be previously found in cancer studies according to the Catalogue Of Somatic Mutations In Cancer (COSMIC) database (http://www.sanger.ac.uk). Only *TP53*, with its well-known role in numerous cancers and *PER1*, which is found to be involved in translocations in leukaemias, are included in the cancer gene census list [13] (http://cancer.sanger.ac.uk/cancergenome/projects/census). A thorough literature search was performed on the 65 genes affected by point mutations. Six genes including *TP53*, *LOXL3*, *ARID1B*, *PAPPA*, *CYP3A43* and *FAT4* were obviously relevant in the context of cancer. Not surprisingly, the tumor suppressor gene *TP53* (17p13.1) is affected by both LOH and a point mutation, that is predicted damaging by SIFT, Polyphen2 and MutationTaster, in all stages of progression. Most noticeable are the frameshift deletion in *LOXL3* which is also hit by both LOH in all samples, and the nonsynonymous mutations in *ARID1B* (6q25.3) and *PAPPA* (9q33.1), which are affected by LOH in all tumor stages but exclusively hit by point mutations in the asynchronous metastasis, suggesting that these genes might be involved in metastatic progression. The mutations are predicted to be deleterious by all three functional prediction scores and reduced expression of the *LOXL3* gene and *PAPPA* gene are found to be significantly associated with shorter recurrence free survival (RFS) with *p*-value 3.4e-7 and *p*-value 1.5e-7, respectively, according to the gene expression data provided by KM Plotter [14] (Kaplan Meier plots for the two genes are shown in Supplementary Figures 5–6). The *CYP3A43* gene (7q22.1) is affected by a point mutation in all tumor steps and in addition LOH in the asynchronous metastasis. The nonsynonymous mutation is predicted to be highly deleterious and gene expression data show a highly significant relationship between low expression of the gene and reduced RFS (* p*-value 1.5e-9) (Kaplan Meier plot is shown in Supplementary Figure 7). The *FAT4* gene is affected by both LOH and a nonsynonymous mutation predicted deleterious exclusively in the metastasis and low expression of the gene is significantly associated with reduced RFS (* p*-value 4.7e-5) (Kaplan Meier plot is shown in Supplementary Figure 8).

### Subclonal copy number events and deep sequencing frequencies of point mutations reveal clonal evolution and suggest the occurrence of catastrophic events

Density plots of mutation frequencies of validated somatic nonsynonymous point mutations, not affected by copy number events, are displayed in Supplementary Figures 9–12. Homogeneity of mutation frequencies within DCIS 1 is seen, supporting that DCIS 1 is monoclonal. Density plots of DCIS 2 and primary tumor illustrate subclonality with two major peaks, again supporting copy number data. The subclonality within the asynchronous metastasis is mostly characterized by the subclonal copy number loss events, but also supported by a few point mutations not affected by concurrent copy number aberrations.

**Figure 6:**
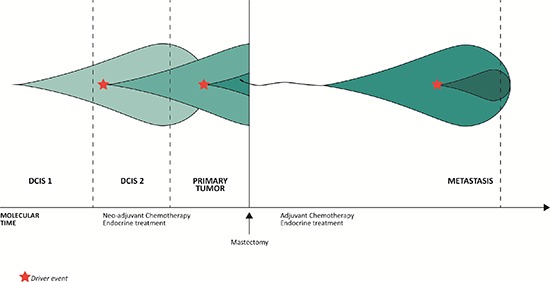
The evolution of clonal populations within the different steps of malignant progression of the studied cancer genome The three vertical lines to the left represent analysis of the three topologically separated tissue samples and the vertical line to the right represent analysis of the metastasis, which is separated from the other malignant steps by both space and time. The increase in color intensity reflects the acquisition of additional somatic events.

Figure 6 displays the clonal evolution of the studied cancer genome in a plot of genetic events in molecular time. A driver event provides a selective advantage for a cell creating a subclone with new, additional mutations, while retaining the old as imprints in the genome, which is in accordance with a model of linear progression. The purification of subclonally occuring somatic aberrations in the primary tumor to complete events of the metastasis provides evidence of a single cell to be ancestor of the metastatic lesion.

## DISCUSSION

We set out to illuminate three aspects of breast cancer progression, the first being the controversy between the two fundamental models of metastatic progression. According to the parallel progression model, the metastasis founder cell disseminates early from the primary tumor, resulting in independent accumulation of genetic events in the metastasis. Concordantly, due to the inherent genomic instability of malignant cells one must also expect further genomic development in the primary tumor from the time point of dissemination of the metastatic cell to the time point of mastectomy. Hence, in order to provide evidence for the parallel progression model, one would expect to see genetic events exclusive to the primary tumor. However, this is not the case in our study. At its simplest, the model of linear progression does not take further genetic evolution of the disseminated cells into account. We find considerable additional genetic evolution in the metastatic lesion. We report a model of linear progression with relatively late dissemination from the primary tumor and additive accumulation of somatic events, also in the late stage of progression leading to genomic discordance between primary tumor and metastasis.

Next, our data are also informative for the phenomenon of clonal diversity within a cancer cell population. A stepwise accumulation of aberrations in several equally competitive lineages would result in a clonally diverse tumor with branching evolution of multiple independent and prominent subclones. A completely monoclonal tumor results if one lineage completely outgrows the others and no additional genetic aberrations conferring a selective advantage in the population are acquired. An intermediate scenario arises if the bulk of the tumor contains a certain set of aberrations and a subclone with additional aberrations, mediating a selective advantage to the progeny, arises relatively late in molecular time allowing the two subpopulations to coexist. Our study reveals the latter model of cancer cell evolution.

The third aspect is the degree of homogeneity within a cancer cell subclone, which addresses the question of stepwise versus catastrophic acquisition of novel events. A stepwise acquisition of somatic events within a lineage would most likely entail a number of consecutive subclones with varying fractions of malignant cells harboring the different aberrations, provided that none of the novel aberrations confer a significant selective advantage allowing it to outperform all other consecutive subclones. If a catastrophic event mediated the selective sweep by creating many aberrations at the same time or in a series of highly compressed events in molecular time, all the cells within the subclone will carry the aberrations. Thus, a density plot with mutational frequencies comprised of two peaks can suggest the appearance of a catastrophic event. The existence of two distinct levels of copy number loss, supported by the allelic fractions in the BAFs, are found in the metastasis revealing that an array of loss events private to the metastasis originates from the same fraction of cells. Hence, copy number data suggests evidence of a catastrophic event resulting in 17 additional loss events in a subpopulation of metastatic cells. Whether a catastrophic driver event comprise a single catastrophic event like chromothripsis or result from several, highly compressed events in molecular time, providing significant selective advantage to the progeny and thus resulting in a homogenous subclone, cannot be concluded from this study.

Naturally, the resolution of the ngCGH assay does not allow identification of very small subclones. Due to the inherent instability of cancer genomes, molecular heterogeneity constantly arises in a cancer cell population; the key factor is whether the new aberrations confer a selective advantage to the progeny. The frequency of a few point mutations drops during progression, suggesting that they are present in the less competitively advantageous clone and some mutations most likely are lost in the metastasis due to copy number loss events.

Molecular heterogeneity already within DCIS supports the idea of clonal selection toward invasive disease, where some malignant cells, in accordance with Darwinian evolution, in addition to the founder genetic aberrations, acquire aberrations enabling them to fulfill the requirements of invasion. This also applies for the later stages of malignant progression. Biological phenomena such as invasiveness, drug resistance and the ability to metastasize constitute evolutionary bottlenecks for the malignant cells, forcing them to acquire new abilities. Our study reveals evolution of copy number events and point mutations during breast cancer progression, a phenomenon influenced by several factors including time, increased genomic instability and selection pressures provided by treatment and endogenous immunological and microenvironmental factors. Clonal heterogeneity has been linked to poor clinical outcome in chronic lymphocytic leukemia [15] and in breast cancer [16]. In our patient only one subclone was found, which gave rise to the metastasis.

This proof of principle study, limited to only one patient, calls for extensions on a larger patient material. Breast cancer is known to display both inter-tumoral and intra-tumoral heterogeneity [17] and thus, the findings of this study are not comprehensively covering evolution of any breast cancer genome.

The studied patient had undergone neo-adjuvant chemotherapy, thus, all tumor samples of this study have survived the selection pressures provided by treatment. A tissue specimen prior to the neo-adjuvant chemotherapy and endocrine therapy might have contained non-resistant tumor clones and the cancer genome of our study likely represents a highly malignant and therapy-resistant cancer as the disease progresses in spite of extensive treatment. Furthermore, one could imagine different genome evolution if additional distant metastases were analyzed from the patient. Significant discordance in estrogen receptor and progesterone receptor status have been reported between different distant breast cancer metastases within the same patient [18].

Patient tailored medicine stresses the therapeutic relevance of uncovering the genomic concordance between a primary breast cancer and its metastases as primary tumors in clinical practice are used as surrogates for systemic disease. This highlights the need for studies elucidating genetic changes during progression of the disease. Relatively few studies have reported genomic copy number evolution during breast cancer progression using comparative genomic hybridization (CGH) [19, 20], arrayCGH [21, 22], multiplex ligation probe amplification (MLPA) [23] and targeted next generation sequencing [24]. Concordant with our results, previous global studies have found relatively similar genetic composition of primary breast cancers and matched metastases, however with some genetic divergence [25, 26].

The genomes of cancer cells acquire extensive genomic alterations due to increasing genomic instability but a significant proportion of these events are merely passenger events that do not provide any selective advantage for the cell. Distinguishing driver mutations and genes from passengers at different steps of malignant progression is the major challenge of cancer genomics, complicated by the fact that the roles of driver and passenger genes may change during progression of the disease [17]. In our study, the most obvious driver gene candidates based on literature search include *TP53, LOXL3*, *ARID1B, PAPPA*, *FAT4* and *CYP3A43*. However, all the altered genes of the studied cancer genome are potential drivers. Genes altered exclusively in the metastasis are potentially new metastasis suppressor genes (MSGs) or genes that orchestrate the expression of several MSGs as recently suggested [27]. However, it must be stressed that copy number gains or losses does not automatically result in altered expression of the affected genes as it is likely that many genes are compensatory repressed or induced either by regulation of transcription factors or epigenetic regulation. Epigenetic changes play key roles in cancer [28] and recently a metastasis-specific methylation signature was reported [29], however, this layer in cancer biology was not included in our study.

In summary, we provide evidence for linear progression of metastatic disease in which dissemination from the primary tumor occurs relatively late in molecular time. Our study reveals common ancestry of the malignant cells and that early acquired copy number aberrations as well as point mutations are retained as imprints in the cancer genome, but also shows substantial acquisition of additional aberrations in the metastasis. We report limited tumor heterogeneity from ongoing clonal linear evolution with continuous positive selection at every stage of malignant progression, where previously acquired aberrations coexist with newly acquired aberrations. The emergence of new aberrations in the metastasis reveal incomplete concordance between early tumor stages and systemic disease and emphasizes the importance of genomic analysis on not only of the primary tumor but also on metastatic tissue at recurrence in order to offer the patients molecularly targeted therapy.

## METHODS

### Patient material

Exome sequencing was performed on successive tumor samples from a 58 year-old breast cancer patient with estrogen receptor positive, HER2-negative, node positive invasive ductal carcinoma, initially treated with five series of neo-adjuvant Cyclophosphamide, Epirubicin and Fluorouracil (CEF). Ductal Carcinoma *in Situ* (DCIS) from two topologically different regions adjacent to the primary tumor and the primary tumor measuring 50 mm were secured during primary surgery and stored at –80°C until sample preparation. Following mastectomy, the patient received four series of Taxotere/Gemcitabine and radiation therapy. In parallel, the patient was initially treated with Tamoxifen and after 2.5 years the endocrine treatment was changed to Anastrozole. In spite of the extensive therapy and ongoing endocrine treatment, the patient experienced recurrence after 4.05 years and later succumbed to the malignant disease. An asynchronous metastasis was biopsied from a contralateral periclavicular lymph node metastasis. Haematoxylin-eosin sections of all tissue samples were reviewed by a certified pathologist ensuring the diagnosis and a content of malignant cells of 75% at minimum. A start amount of 20–30 mg fresh frozen tissue (asynchronous metastasis 5 mg) was used for the purification process. Tissue disruption and homogenization was performed using TissueLyser (Qiagen) and purification of DNA was performed using AllPrep DNA/RNA Mini Kit (Qiagen). The primary tumor and matched normal tissue were stored as formalin-fixed paraffin-embedded (FFPE) tissue. The FFPE blocks were cut in 30–40 sections of 10 μm and DNA extracted using AS1000 Maxwell 16 (Promega, USA).

The patient consented to participate in the study and for the data to be published. The study was approved by the Ethical Committee of Region Syddanmark and notified to the Danish Data Protection Agency.

### Library construction and exome sequencing

Exome enrichment was performed with Illumina's TruSeq DNA Sample Preparation and sequenced on the Illumina HiSeq 1500 platform. FASTQ files were aligned to the human reference genome GRCh37 (feb.2009) using the Novoalign v. 3 algorithm (http://www.novocraft.com) at default parameters. Removal of duplicate reads, recalibration and local realignment around indels was performed using Best Practices pipeline v. 2.7 [30]. The result was mean coverage rates in the exome region between 89 x and 148 x (Supplementary Table 1).

### Copy number profiling and correction for aneuploidy

The Nexus 7.5 software (BioDiscovery) was applied for the detection of somatic copy number events using the ngCGH software (http://github.com/seandavi/ngCGH), in which the processing of tumor and process-matched normal sequencing BAM files computes a pseudo-CGH using simple coverage counting on the tumor reads relative to normal reads. Each window is defined by 1000 reads in the normal tissue BAM file. Within each defined genomic window the number of reads in the tumor is quantified and a ratio is made between the number reads in the tumor and the number of reads in the normal. Finally, a Log2 transformation is applied to each ratio and the entire vector of the results is then centered by subtracting the median. A diploid region (ratio 1:1) results in a Log2 ratio of 0 and the probes are placed at baseline. A single copy gain in the tumor sample (ratio 3:2) results in a Log2 ratio of 0.58, while a heterozygote loss in the tumor sample (ratio 1:2) results in a Log2 ratio of –1. Naturally, admixture of normal cells in the sequenced cancer sample compresses the Log2 ratio towards the baseline. The ngCGH software applies Fast Adaptive States Segmentation Technique (FASST2) segmentation to make calls. The minimum number of probes per call was set to 10. All other parameters were run by default. Cancer sample aneuploidy may introduce bias in establishing the Log2 Ratio baseline for copy number calling. True diploid regions in the cancer sample were detected, displaying BAF around 0.5, and the Log2 ratio baseline were adjusted according to these.

The primary tumor DNA in this study originates from FFPE tissue, a storage form known for posing technical challenges. We found the noise introduced by the formalin fixation in the ngCGH assay to be reduced by matching the FFPE tissue derived sequencing data with normal sequencing data also originating from FFPE tissue.

To supplement the computed ngCGH Log2 ratios, which constitute genomic quantity measurements, combined files were created, adding B Allele Frequencies (BAFs) for the tumor sample to be displayed in a panel below. The BAFs reveal the allele distribution between the reference allele (A allele) and the alternative allele (B allele) of the tumor sample and is calculated as

$$\text{BAF}=\frac{B\text{ allele frequency}}{A+B\text{ allele frequency}}.$$

The BAFs for the ngCHG plots included only germline heterozygote positions with known SNPs, annotated by dbSNP (version 135), and covered by at least 30 × in both tumor and normal sample. Therefore, in the case of no copy number event in the tumor sample, the BAF in this region is 0.5. If all the sequenced cells of a region have lost one of the two alleles, the BAFs would split out to 0 and 1. The deviation from a 0/1 split out of BAFs in a complete heterozygote loss event reflects the degree of normal cell admixture in the tumor sample. A subclonal event results in intermediate BAFs as only a fraction of cells have lost one of the alleles.

### Point mutation and indel calling

The BAFs reported from the somatic point mutation and indel calling are also calculated as

$$\text{BAF}=\frac{B\text{ allele frequency}}{A+B\text{ allele frequency}}.$$

and in this context the BAF depicts the percentage of somatic mutation alternative reads in the tumor sample. Variant calling was performed using Varscan 2 [31] version 2.3.6. Included were positions with normal tissue read depth of min. 10 and normal tissue homozygote for the reference allele defined by BAF < 0.02. Only positions with min. 3 alternative reads and BAF > 0.15 in one of the tumor samples were included and mutation noted when BAF > 0.05. The variants were annotated with Annovar [32]. Known SNPs with an allele frequency > 1% were excluded. Validation with deep sequencing using SureSelect (Agilent) target enrichment resulted in a mean coverage of 377 ×. The functional significance of the validated nonsynonymous SNVs were assessed by the functional prediction algorithms SIFT [33], PolyPhen2 [34] and Mutation Taster [35]. Gene expression levels from breast cancer studies with outcome data were utilized using the Kaplan Meier plotter online tool (http://www.kmplot.com) [14] in order to evaluate the effect of altered gene transcription levels on recurrence free survival.

## SUPPLEMENTARY FIGURES AND TABLES










