# Supplementary material for: Clonal expansion and linear genome evolution through breast cancer progression from pre-invasive stages to asynchronous metastasis
Source: Oncotarget. 2015 Jan 29;6(8):5634–49. doi: 10.18632/oncotarget.3111 (PMC4467391; doi:10.18632/oncotarget.3111)
Supplement: Supplementary file 1 [file oncotarget-06-5634-s001.pdf]

SUPPLEMENTARY FIGURES AND TABLES

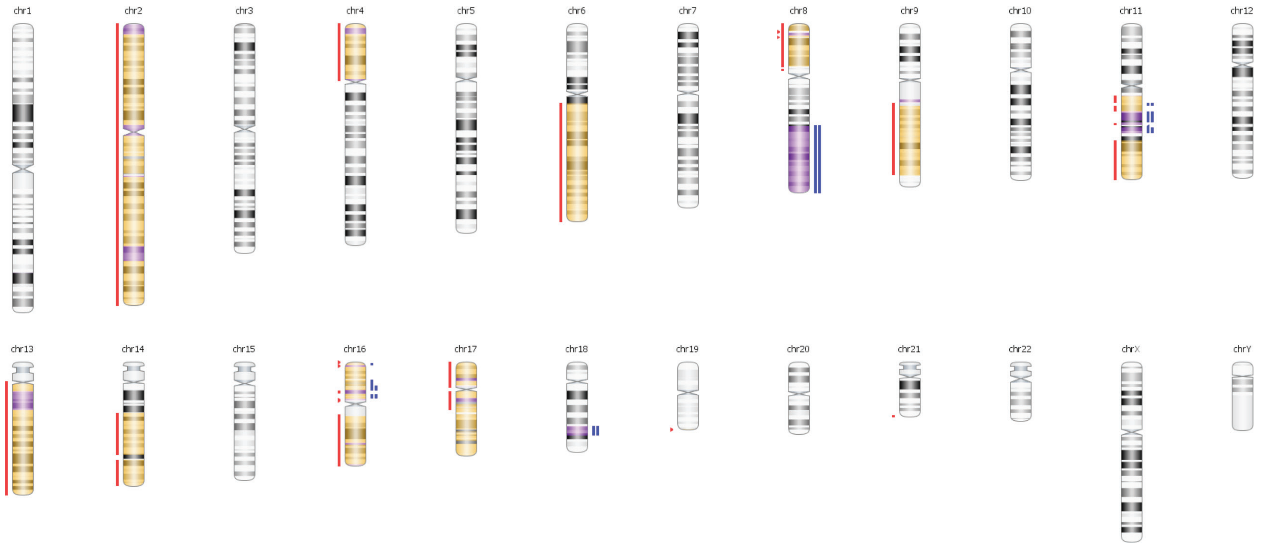

Supplementary Figure 1: Overview of copy number aberrations in DCIS 1.

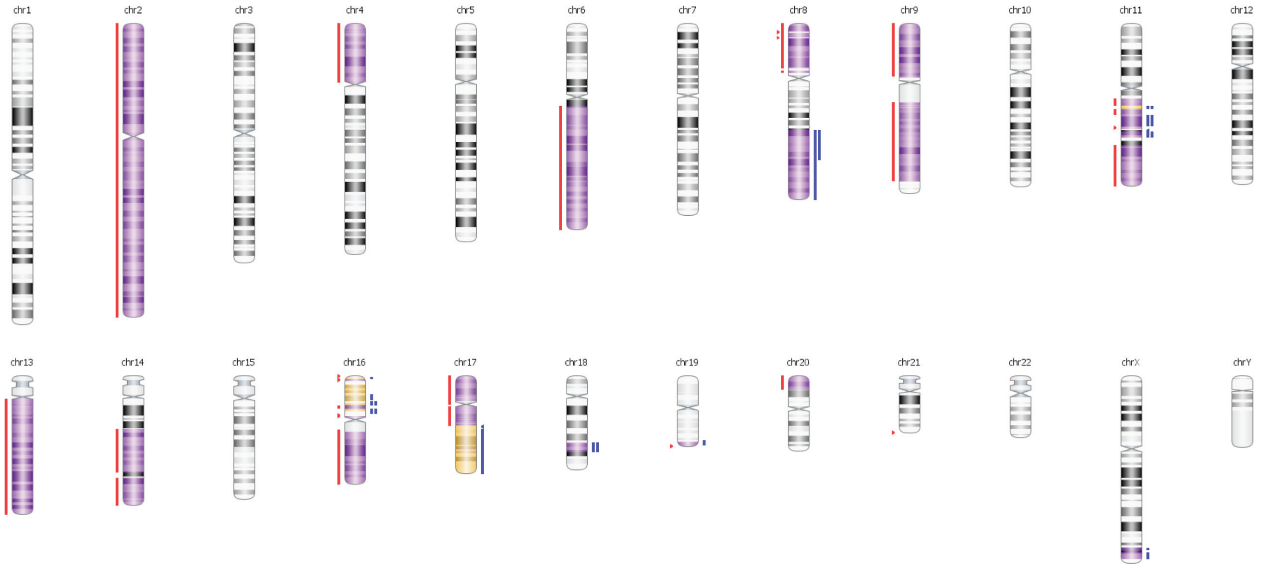

Supplementary Figure 2: Overview of copy number aberrations in DCIS 2.

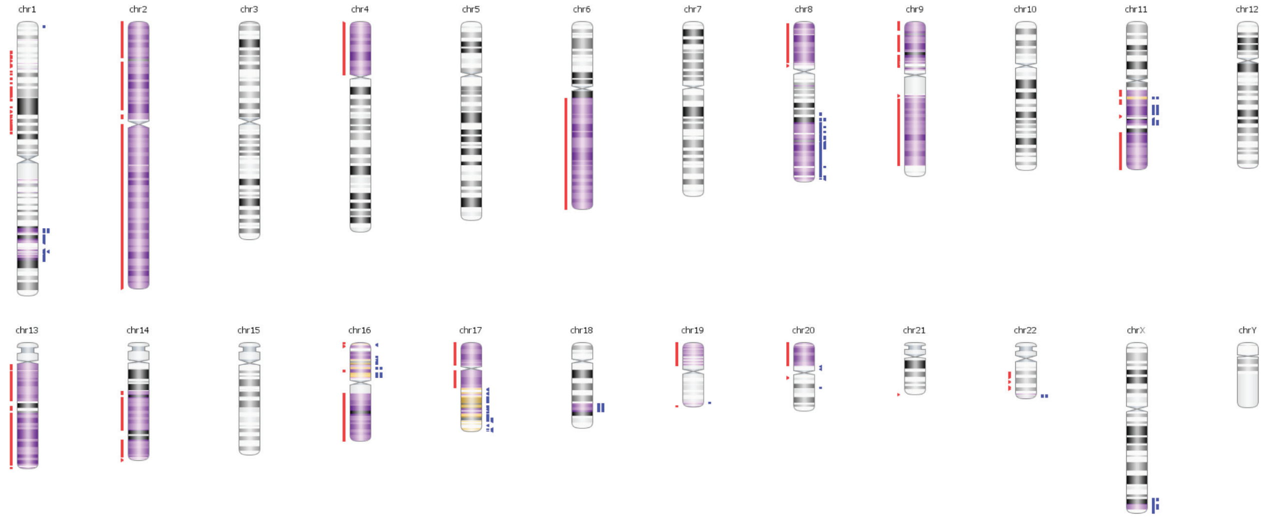

Supplementary Figure 3: Overview of copy number aberrations in the primary tumor.

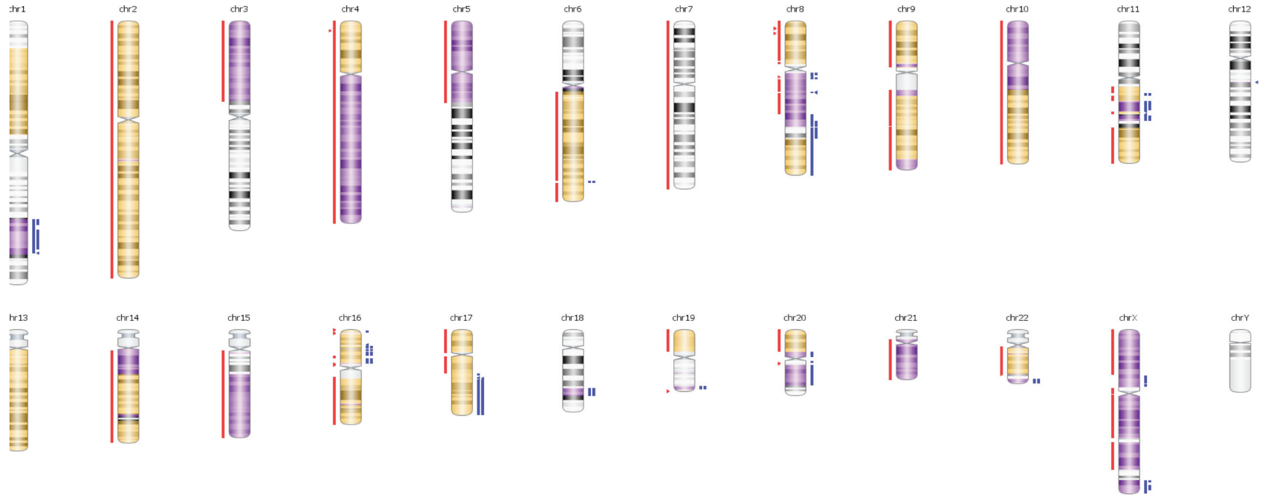

Supplementary Figure 4: Overview of copy number aberrations in the asynchronous metastasis.

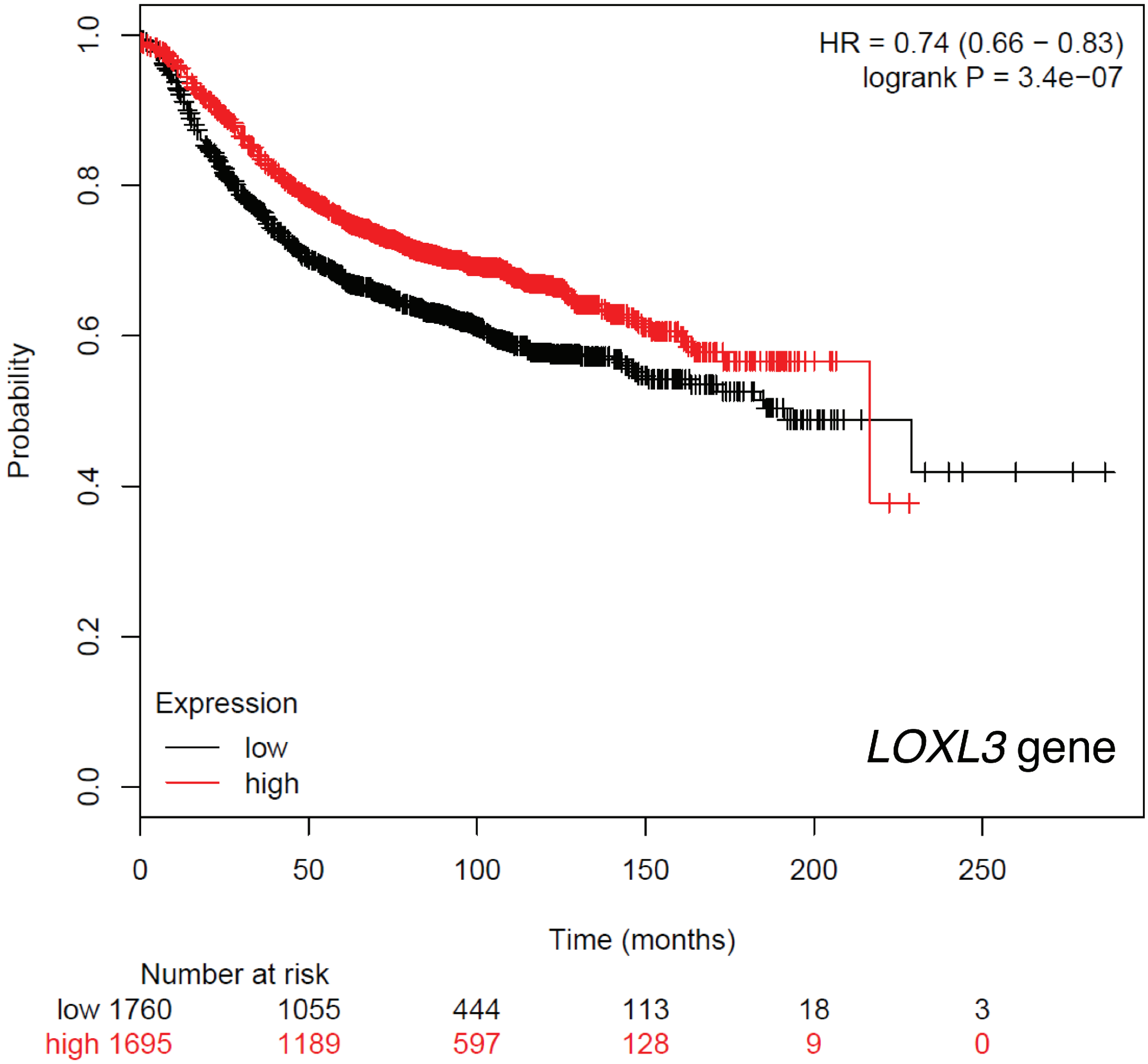

Supplementary Figure 5: Kaplan Meier plot based on gene expression data for the *LOXL3* gene.

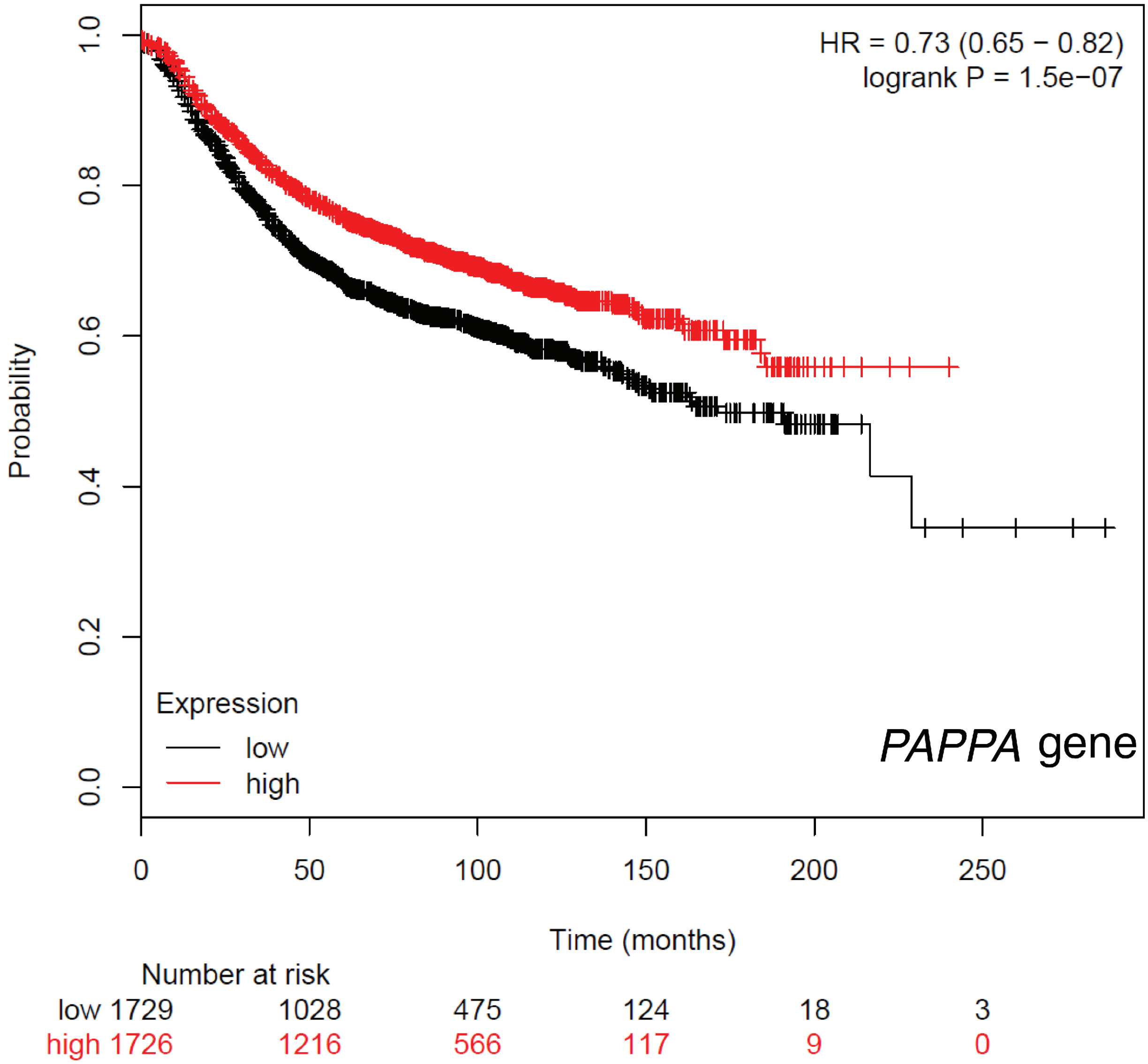

Supplementary Figure 6: Kaplan Meier plot based on gene expression data for the *PAPPA* gene.

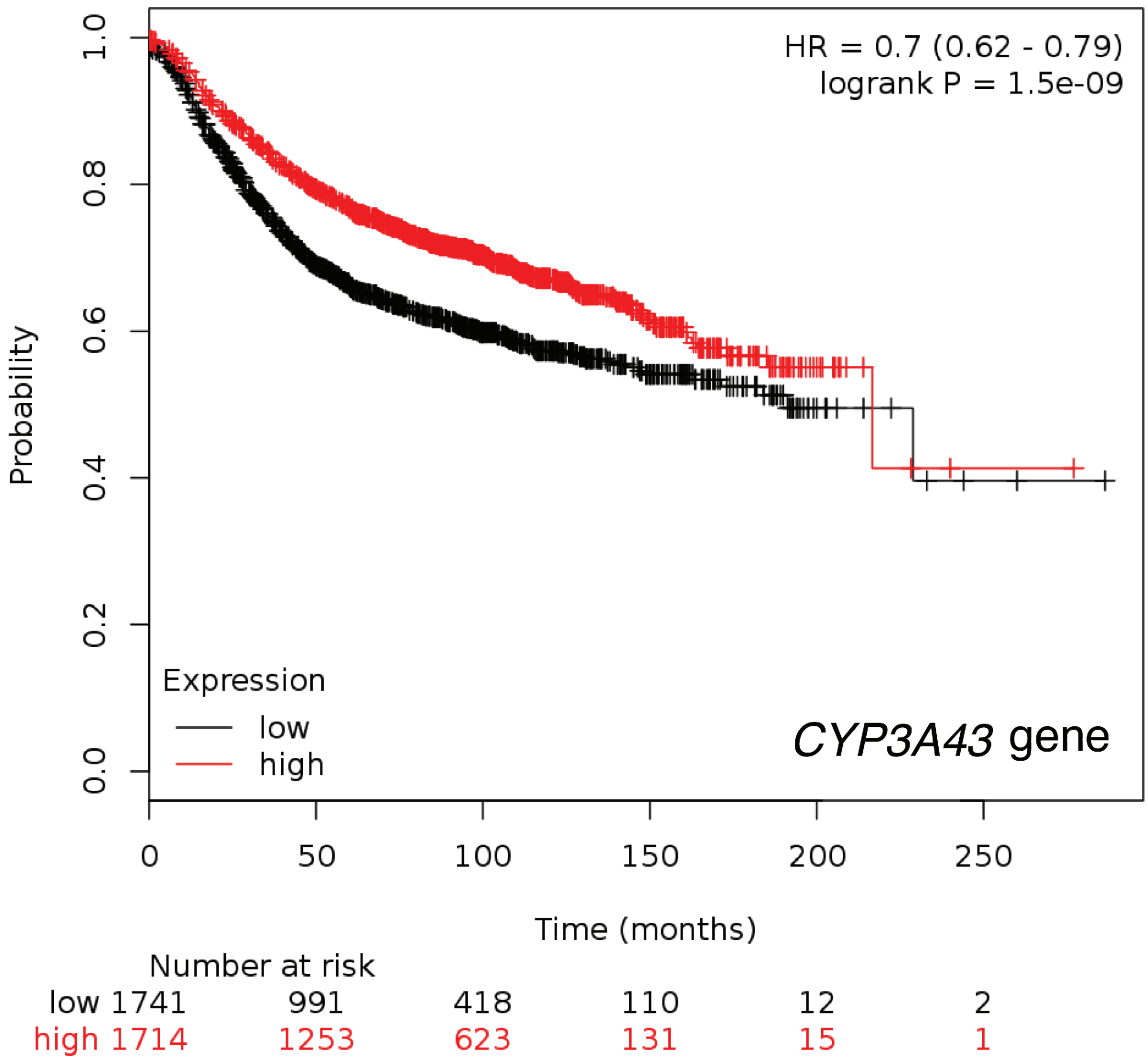

Supplementary Figure 7: Kaplan Meier plot based on gene expression data for the *CYP3A43* gene.

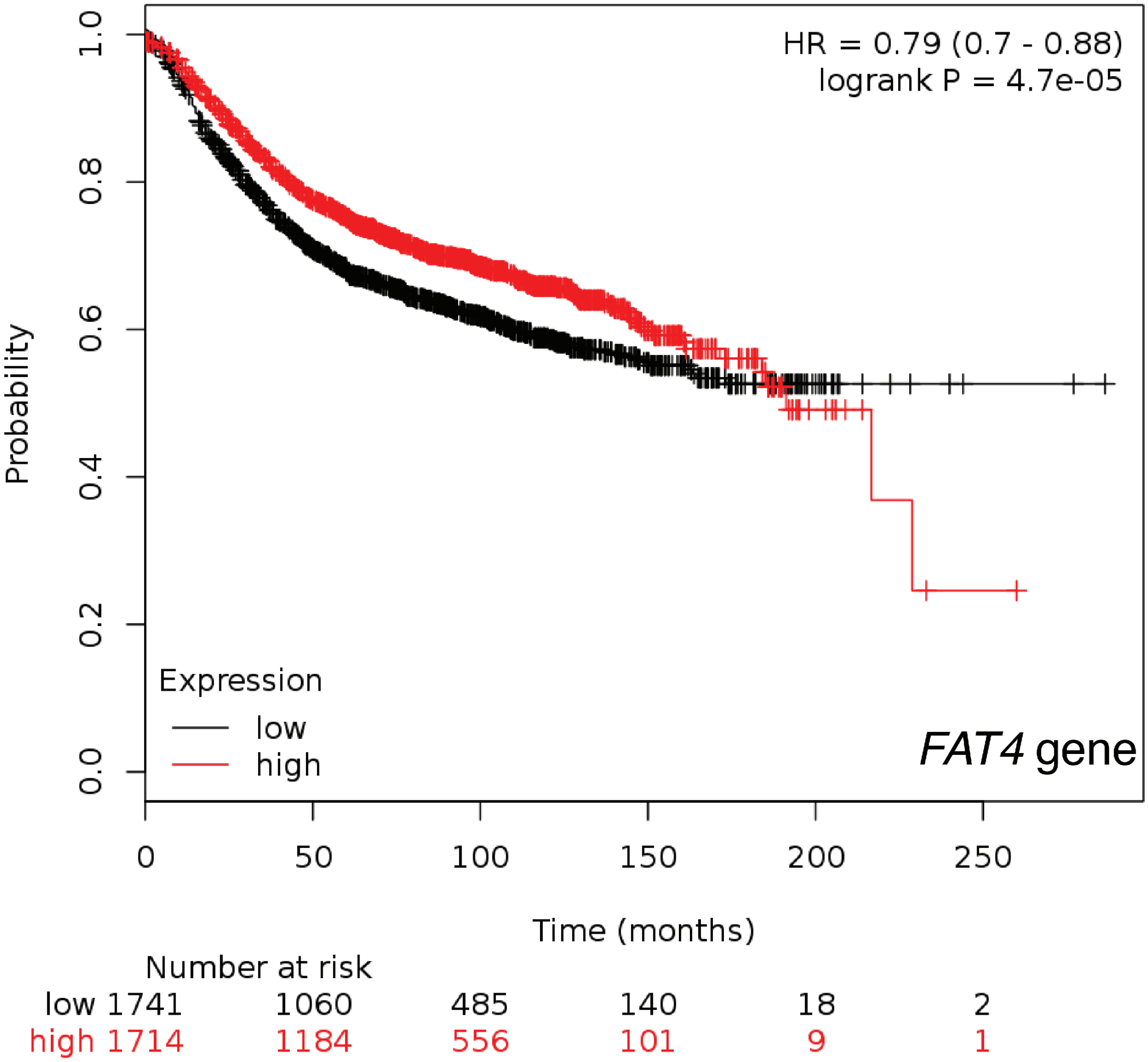

Supplementary Figure 8: Kaplan Meier plot based on gene expression data for the *FAT4* gene.

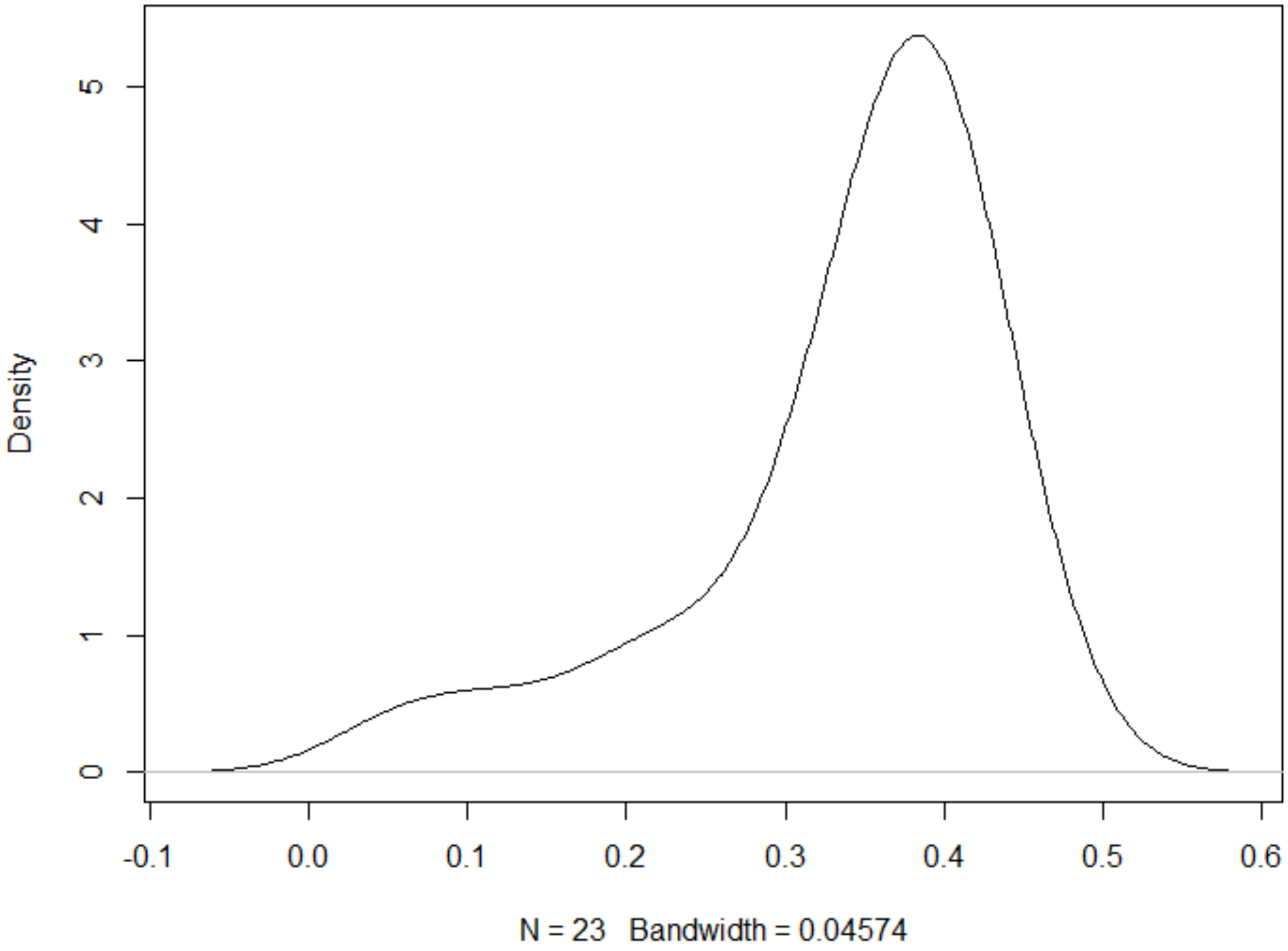

Supplementary Figure 9: Density plot DCIS 1.

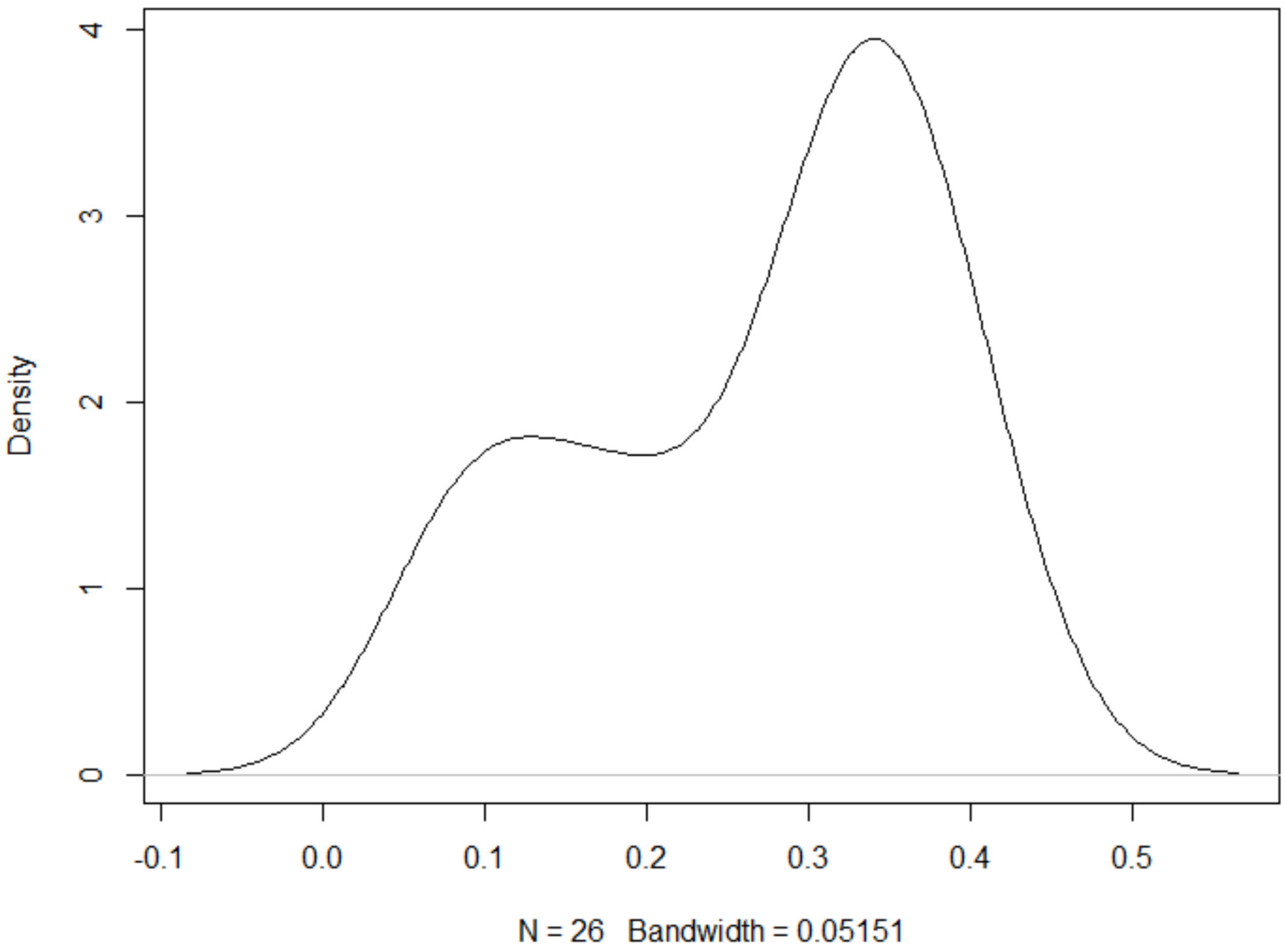

Supplementary Figure 10: Density plot DCIS 2.

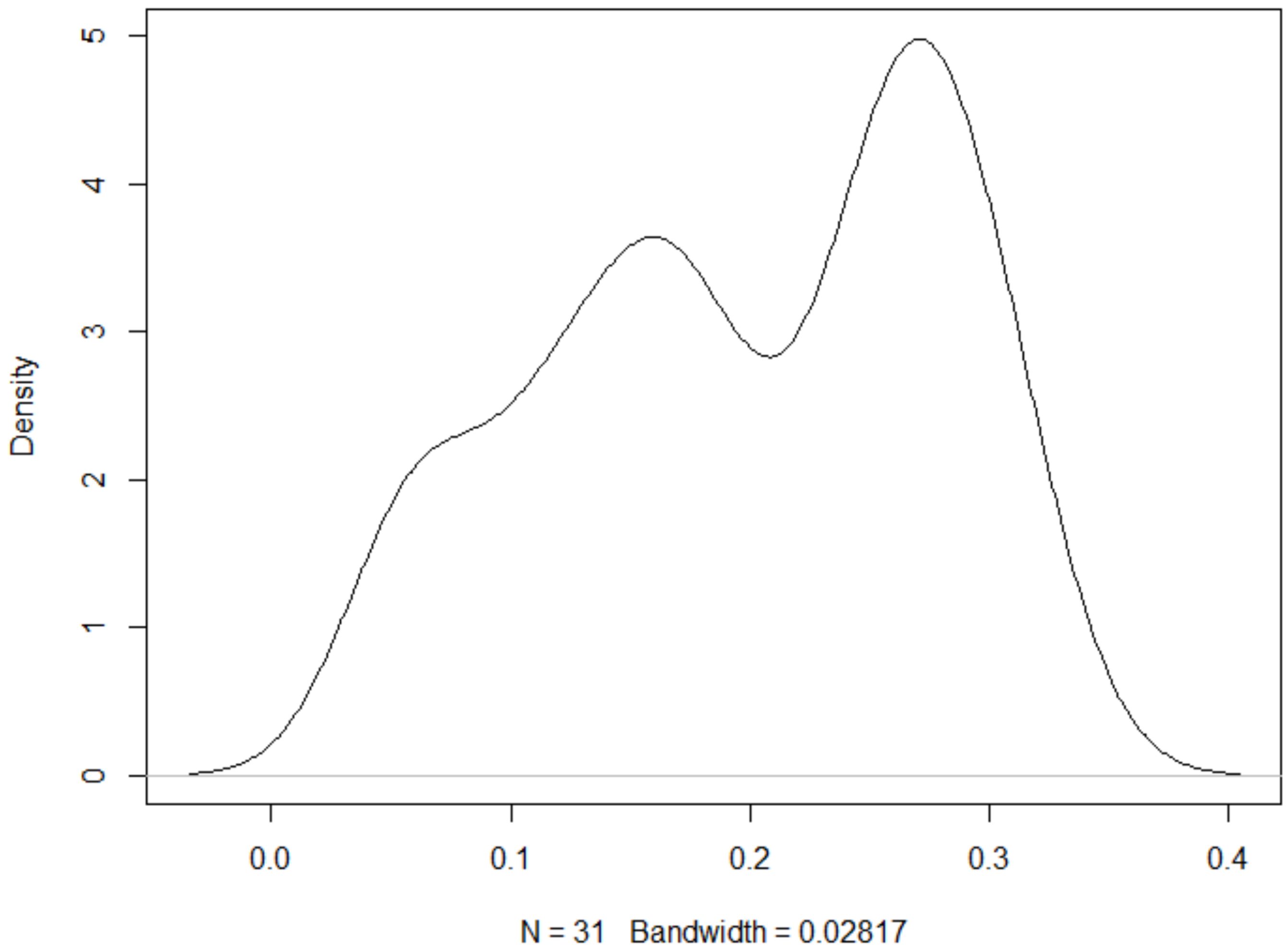

Supplementary Figure 11: Density plot primary tumor.

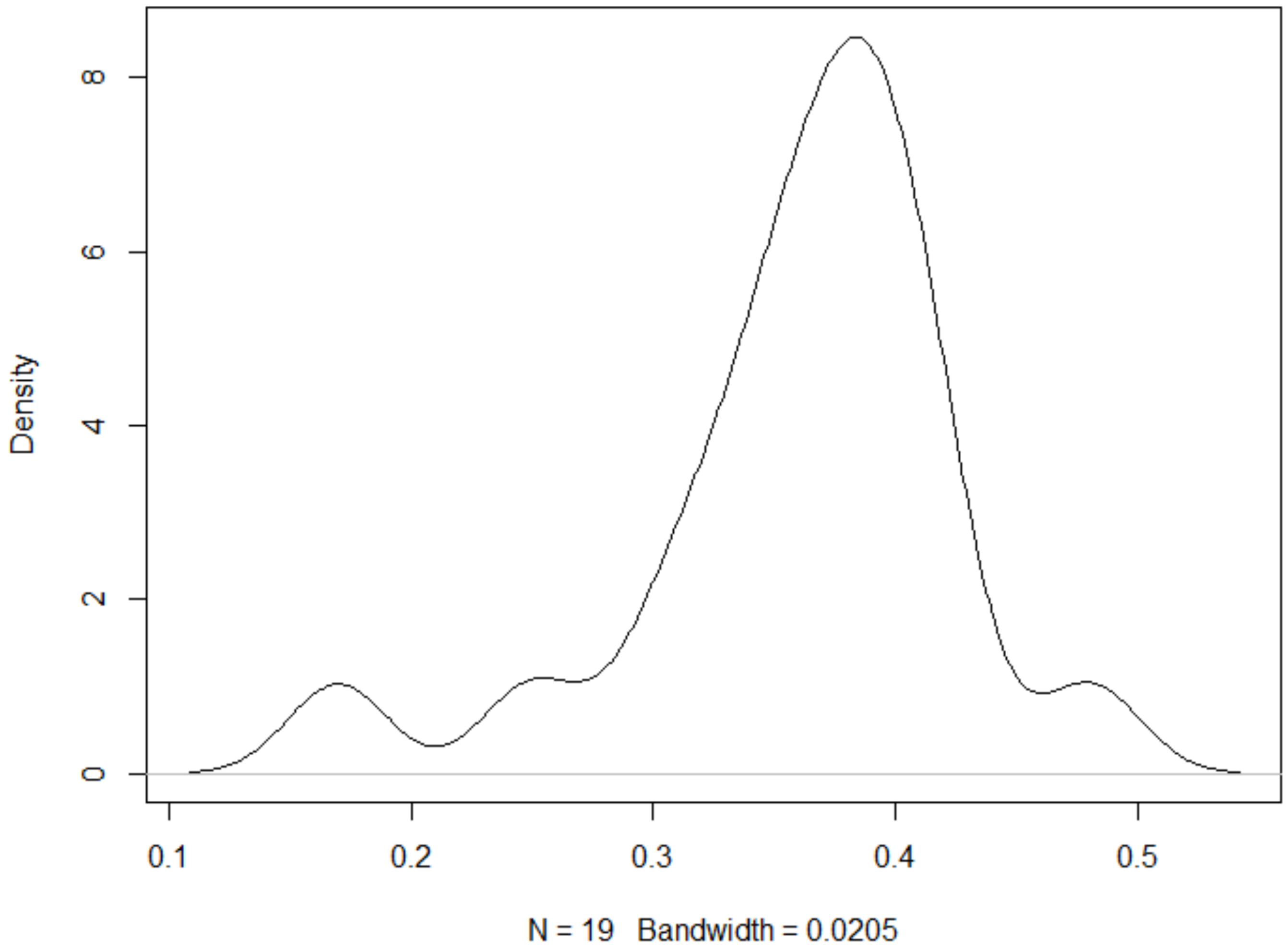

Supplementary Figure 12: Density plot asynchronous metastasis.

Supplementary Table 1: Exome sequencing data

Sample	# Reads	Raw yield GB	% bases > Q30	Yield > Q30 GB	% duplicate reads	# Usable bases	On target bases	On target %	Mean coverage
**DCIS 1**	243928670	24,63	84,9	20,91	40,8	14584983040	6128204906	42,0	98,7
**DCIS 2**	215827498	21,79	84,6	18,43	38,1	13493319350	5541145580	41,0	89,2
**Primary tumor (FFPE)**	157726216	15,93	91,5	14,59	39,2	9684596620	4610528338	47,6	74,2
**Asynchronous metastasis**	268675802	27,13	85,0	23,06	41,8	15793300990	6873687766	43,5	110,7
**Process matched normal tissue (fresh frozen)**	223279458	22,55	85,7	19,32	37,6	14071964560	5971643534	42,4	96,1
**Matched normal tissue (FFPE)**	265430620	26.79	92.8	24.87	30.5	18627919230	9232655487	49,5	148,6

Supplementary Table 2: Copy number evolution of the studied cancer genome with specified fractions of aberration types

Supplementary Table 3: Point mutations with B Allele Frequencies derived from the exome sequencing data.

Gene	Position	Mutation type	DCIS 1	DCIS 2	Primary tumor	Asynchronous metastasis
TP53	17p13.1	nonsynonymous SNV	0.63	0.53	0.52	0.76
LOXL3	2p13.1	frameshift deletion	0.80	0.46	0.43	0.76
SMG6	17p13.3	nonsynonymous SNV	0.08	0.36	0.25	0.69
NPAS2	2q11.2	nonsynonymous SNV	0.76	0.59	0.44	0.74
DENND6A	3p14.3	nonsynonymous SNV	0.45	0.40	0.15	0.39
GPAT2	2q11.1	nonsynonymous SNV	0.00	0.05	0.00	0.84
FKBP15	9q32	nonsynonymous SNV	0.00	0.02	0.17	0.78
TLE4	9q21.31	nonsynonymous SNV	0.00	0.00	0.09	0.62
EXOC3L1	16q22.1	nonsynonymous SNV	0.00	0.00	0.00	0.65
ARID1B	6q25.3	nonsynonymous SNV	0.00	0.00	0.00	0.44
NLK	17q11.2	nonsynonymous SNV	0.00	0.00	0.00	0.35
PAPPA	9q33.1	nonsynonymous SNV	0.00	0.00	0.00	0.31
OR1K1	9q33.2	nonsynonymous SNV	0.00	0.00	0.00	0.38
NBAS	2p24.3	nonsynonymous SNV	0.00	0.00	0.00	0.38
ANKRD20A4	9q21.11	nonsynonymous SNV	0.00	0.00	0.00	0.16
NCKAP5	2q21.2	Splicing	0.00	0.00	0.00	0.35
IRS1	2q36.3	nonsynonymous SNV	0.10	0.20	0.00	0.07
CEP164	11q23.3	nonsynonymous SNV	0.00	0.17	0.00	0.07
FRYL	4p11	nonsynonymous SNV	0.09	0.17	0.05	0.05
PER1	17p13.1	nonsynonymous SNV	0.00	0.17	0.00	0.00
DOCK6	19p13.2	nonsynonymous SNV	0.00	0.00	0.00	0.42
ACTG2	2p13.1	nonsynonymous SNV	0.58	0.20	0.00	0.00
PLAA	9p21.2	nonsynonymous SNV	0.42	0.22	0.37	0.79
HFM1	1p22.2	nonsynonymous SNV	0.35	0.34	0.30	0.02
BPIFC	22q12.3	nonsynonymous SNV	0.35	0.34	0.25	0.00
POM121L12	7p12.1	nonsynonymous SNV	0.54	0.08	0.00	0.00
SLCO5A1	8q13.3	nonsynonymous SNV	0.36	0.09	0.14	0.00
MOSPD2	Xp22.2	nonsynonymous SNV	0.37	0.16	0.03	0.00
WDFY4	10q11.23	nonsynonymous SNV	0.07	0.22	0.22	0.72
ZDHHC9	Xq26.1	nonsynonymous SNV	0.37	0.16	0.31	0.47
ZNF716	7p11.2	nonsynonymous SNV	0.37	0.33	0.35	0.34
MUC12	7q22.1	Nonsynonymous SNV	0.11	0.13	0.00	0.14
CNOT4	7q33	nonsynonymous SNV	0.39	0.29	0.29	0.32
CYP3A43	7q22.1	nonsynonymous SNV	0.39	0.30	0.23	0.30
ANKRD28	3p25.1	Splicing	0.06	0.17	0.17	0.38
DRP2	Xq22.1	nonsynonymous SNV	0.02	0.22	0.25	0.55

(*Continued*)

Gene	Position	Mutation type	DCIS 1	DCIS 2	Primary tumor	Asynchronous metastasis
NYNRIN	14q12	stopgain SNV	0.02	0.20	0.20	0.30
XKR4	8q12.1	stopgain SNV	0.01	0.02	0.18	0.53
MAN1B1	9q34.3	nonsynonymous SNV	0.00	0.01	0.16	0.48
AGAP6	10q11.23	nonsynonymous SNV	0.00	0.03	0.16	0.54
MED12	Xq13.1	stopgain SNV	0.12	0.17	0.33	0.04
GLT6D1	9q34.3	nonsynonymous SNV	0.17	0.08	0.00	0.22
IGDCC4	15q22.31	nonsynonymous SNV	0.18	0.19	0.00	0.16
DACH2	Xq21.2	nonsynonymous SNV	0.00	0.00	0.00	0.31
FAT4	4q28.1	nonsynonymous SNV	0.00	0.00	0.00	0.24
LBX1	10q24.31	nonsynonymous SNV	0.00	0.00	0.00	0.23
PRAF2	Xp11.23	nonsynonymous SNV	0.00	0.00	0.00	0.23
EIF2D	1q32.1	nonsynonymous SNV	0.02	0.02	0.17	0.26
SLC16A6	17q24.2	nonsynonymous SNV	0.00	0.01	0.20	0.16
SPATC1	8q24.3	nonsynonymous SNV	0.08	0.20	0.06	0.09
ZNFX1	20q13.13	nonsynonymous SNV	0.00	0.00	0.00	0.38
COIL	17q22	nonsynonymous SNV	0.16	0.14	0.00	0.10
GRK6	5q35.3	nonsynonymous SNV	0.21	0.18	0.13	0.00
KIAA1033	12q23.3	nonsynonymous SNV	0.45	0.29	0.36	0.49
TMEM57	1p36.11	nonsynonymous SNV	0.27	0.33	0.29	0.47
OR9K2	12q13.2	nonsynonymous SNV	0.28	0.42	0.32	0.45
S100A5	1q21.3	nonsynonymous SNV	0.40	0.38	0.31	0.42
ASH1L	1q22	nonsynonymous SNV	0.30	0.29	0.23	0.42
C3orf27	3q21.3	nonsynonymous SNV	0.52	0.38	0.24	0.41
HHIPL2	1q41	stopgain SNV	0.36	0.38	0.34	0.41
FHOD3	18q12.2	nonsynonymous SNV	0.36	0.30	0.42	0.38
CNTN5	11q22.1	nonsynonymous SNV	0.45	0.40	0.24	0.28
ZBBX	3q26.1	nonsynonymous SNV	0.44	0.28	0.21	0.22
PCDHGA9	5q31.3	nonsynonymous SNV	0.04	0.19	0.19	0.37
ZNF706	8q22.3	nonsynonymous SNV	0.12	0.16	0.05	0.04
PITPNM2	12q24.31	nonsynonymous SNV	0.00	0.04	0.19	0.54
KCNQ2	20q13.33	nonsynonymous SNV	0.00	0.02	0.13	0.54
MYLK	3q21.1	nonsynonymous SNV	0.00	0.00	0.05	0.42
LRRC52	1q24.1	nonsynonymous SNV	0.00	0.01	0.19	0.36
TMTC1	12p11.22	nonsynonymous SNV	0.00	0.00	0.24	0.54
SHANK3	22q13.33	nonsynonymous SNV	0.00	0.00	0.00	0.44
VCAN	5q14.3	nonsynonymous SNV	0.00	0.00	0.00	0.35
C6orf52	6p24.2	nonsynonymous SNV	0.00	0.00	0.00	0.22

Supplementary Table 4: Elaborated version of Figure 3. Validated point mutations with B Allele Frequencies combined with copy number events within each step of malignant progression

Supplementary Table 5: Genes affected by copy number gain events exclusive to the asynchronous metastasis

Supplementary Table 6: Genes affected by copy number loss events exclusive to the asynchronous metastasis
